# Integrated Antioxidants, Nanoparticle, and Antifreeze Protein Strategies Synergistically Enhance Cryotop Vitrification Outcomes of Porcine Parthenogenetic Embryos

**DOI:** 10.3390/antiox14121412

**Published:** 2025-11-26

**Authors:** Jesse Oluwaseun Ayantoye, Baigao Yang, Jianhua Dong, Xiaoyi Feng, Muhammad Shahzad, Hubdar Ali Kolachi, Pengcheng Wan, Hongmei Pan, Xueming Zhao

**Affiliations:** 1 Institute of Animal Sciences (IAS), Chinese Academy of Agricultural Sciences (CAAS), No. 2 Yuanmingyuan Western Road, Haidian District, Beijing 100193, China; 2023y90100045@caas.cn (J.O.A.); 82101211205@caas.cn (B.Y.);; 2 State Key Laboratory of Sheep Genetic Improvement and Healthy Breeding, Institute of Animal Husbandry and Veterinary Sciences, Xinjiang Academy of Agricultural and Reclamation Sciences, Shihezi 832000, China; 3 Chongqing Academy of Animal Sciences, Chongqing 402460, China

**Keywords:** Cryotop vitrification, porcine embryos, berberine, melatonin, Fe_3_O_4_ nanoparticles, antifreeze protein I, oxidative stress, apoptosis

## Abstract

Porcine embryo cryopreservation remains challenging due to high lipid content, oxidative stress, and ice recrystallization that compromise post-thaw survival and developmental competence. We evaluated an integrated vitrification approach combining antioxidants (berberine, melatonin), iron oxide (Fe_3_O_4_) nanoparticles, and antifreeze protein I (AFP I) with post-thaw interventions (glutathione and zona pellucida digestion) to synergistically improve cryosurvival and developmental competence of porcine parthenogenetic embryos. In vitro-matured parthenogenetic embryos were vitrified on Cryotop using a protocol that included berberine and melatonin in embryo culture, Fe_3_O_4_ nanoparticles and AFP I in cryoprotectant solutions, and post-warming treatment with glutathione and a brief zona pellucida digestion. Survival, hatching, adenosine triphosphate (ATP) content, reactive oxygen species (ROS) levels, cytoskeletal integrity, and the expression of *BAX*, *BCL2*, *OCT4*, and *SOX2* genes were measured. Both the dual antioxidant (berberine + melatonin) and nanoparticle + AFP interventions produced greater improvements than individual additives. Fully integrating all components yielded the highest post-thaw viability, with ~94% survival and ~90% hatching, values statistically equivalent to those of fresh embryos. Treated embryos also showed significantly higher ATP levels, lower ROS accumulation (approaching levels in fresh embryos), and preserved microtubule structure (~91% normal). Vitrification alone upregulated *BAX* and downregulated *BCL2*, *OCT4*, and *SOX2*, whereas the integrated protocol restored their expression levels to near control levels. This multi-component antioxidant, nanoparticle, antifreeze strategy synergistically enhances the cryotolerance and developmental competence of vitrified porcine embryos by mitigating oxidative stress and cryoinjury. Post-thaw viability and molecular markers were restored to near-fresh conditions, demonstrating a promising approach to improve embryo cryopreservation outcomes in swine and potentially other species.

## 1. Introduction

Cryopreservation of porcine embryos holds significant promise for agriculture, conservation, and biomedical research [1,2]. In swine production, embryo banking accelerates genetic improvement and enables secure international exchange of germplasm, while reducing the need for live animal transport [3]. Long-term storage also helps conserve rare breeds and safeguard genetic diversity [4,5]. In biomedical contexts, pigs are increasingly crucial as disease models and potential organ donors, making efficient preservation of genetically engineered embryos essential [6,7]. However, progress in porcine embryo cryopreservation has been hindered by species-specific challenges, particularly the high cytoplasmic lipid content, which renders embryos susceptible to chilling injury [8]. Early attempts at slow freezing yielded poor and inconsistent survival rates, and frozen porcine embryos remained rarely used in practice [1,2,9]. During cooling and warming, lipid phase transitions destabilize membranes and organelles [10,11], while ice crystal formation, osmotic stress, and thermal shock cause cytoskeletal disruption and oxidative imbalance [12,13]. Elevated ROS and apoptosis further compromise developmental potential [14,15], which explains why porcine embryos consistently survive cryopreservation at significantly lower rates than those of other livestock species. These limitations have driven the development of vitrification-based approaches, which offer a more effective strategy for overcoming species-specific barriers to porcine embryo cryopreservation.

Vitrification has emerged as the preferred approach to overcoming these species-specific limitations [16,17]. Unlike slow freezing, vitrification employs high concentrations of cryoprotectants and ultrarapid cooling, transforming water into an amorphous glass and preventing ice crystal formation [18]. The adoption of vitrification, particularly through open micro-scale devices such as the Cryotop system, has markedly improved post-thaw viability [19]. Cryotop vitrification can yield survival rates exceeding 95%, even in large batches of embryos, and outcomes comparable to fresh embryos [20,21,22]. Recent advances have therefore positioned vitrification, especially Cryotop and related open devices, as the standard for porcine embryo cryopreservation [1,19,23]. For the swine industry, the adoption of embryo vitrification represents a significant advance in reproductive management [3,24,25,26]. Nonetheless, even vitrified embryos can experience subtle cellular stress and exhibit reduced in vivo developmental rates; thus, there is interest in further optimizing protocols to mitigate cryoinjury.

One promising approach to improving cryo-survival is to incorporate cryoprotective additives that target specific injury pathways [27]. In particular, antioxidant compounds, nanoparticles, and antifreeze proteins have been investigated for their potential to mitigate cryodamage [28]. Berberine (BER), a natural isoquinoline alkaloid, is one notable antioxidant under investigation in this context [29]. BER can scavenge ROS and bolster cellular antioxidant defenses. It has demonstrated cytoprotective and anti-apoptotic effects in various cell types [30]. For example, in mammalian oocytes, low micromolar concentrations of BER have been reported to decrease lipid peroxidation and apoptosis, thereby enhancing developmental competence after vitrification [29]. This finding suggests that berberine could similarly mitigate oxidative stress in cryopreserved pig embryos, which often suffer ROS-induced damage during warming. Another antioxidant of interest is melatonin, which has been shown to improve in vitro maturation of porcine oocytes, increase glutathione content, decrease ROS accumulation, enhance mitochondrial distribution, and promote cleavage and blastocyst formation in parthenogenetically activated embryos [31]. Additionally, melatonin enhances porcine embryo development by activating the Nrf2/ARE signaling pathway, thereby providing further antioxidant protection and supporting embryonic competence [32]. Collectively, these findings suggest that melatonin exerts its effects through both receptor-mediated and redox-regulatory mechanisms to enhance embryo resilience to cryoinjury.

Another promising additive is magnetite (Fe_3_O_4_) nanoparticles, which possess unique thermal and biochemical properties [33]. As superparamagnetic particles, Fe_3_O_4_ nanoparticles can enhance heat transfer during warming. They may also interact with plasma membranes to reinforce structural stability [34]. Abbasi et al. (2021) [33] reported that incorporating Fe_3_O_4_ nanoparticles into the vitrification solution protected mouse oocytes from cryodamage, leading to improved nuclear maturation and blastocyst development compared to conventional vitrification. The treated oocytes exhibited reduced ultrastructural disruption and lower rates of apoptosis, suggesting that the nanoparticles stabilized organelles and cell membranes during cryopreservation. Incorporating such nanoparticles into pig embryo vitrification media might similarly protect embryos against thermal and mechanical stresses.

Additionally, AFPs effectively lower the freezing point of water and limit the size of ice crystals, thereby preventing ice-induced cell dehydration and membrane rupture [35]. Even when bulk ice formation is avoided during cryopreservation, small ice nuclei or devitrification events during warming can still cause damage. AFPs can suppress these microscopic ice-related threats [36,37,38]. Supplementing cryopreservation protocols with AFPs has shown beneficial effects in various scenarios [39]. For instance, adding AFP I to a slow-freezing medium improved the post-thaw survival and hatching rates of sheep embryos [40]. Similarly, an insect-derived AFP has been shown to enhance post-vitrification development of bovine and ovine embryos [41]. Although the effectiveness of AFPs depends on their type, concentration, and usage protocol, their protective mechanism, blocking ice crystal damage, is highly relevant to lipid-rich porcine embryos [35]. AFPs may also interact with cell membranes to stabilize them at low temperatures, providing an additional layer of protection beyond ice inhibition [42]. Thus, AFPs serve as potent cryoprotectants by blocking ice crystal damage and stabilizing membranes, thereby enhancing embryo survival and development after thawing.

Given the multifactorial nature of cryoinjury in porcine embryos, employing a combination of protective agents is an attractive strategy. Berberine, melatonin, Fe_3_O_4_ nanoparticles, and AFP I each address distinct injury pathways, including oxidative stress, mitochondrial dysfunction, physicochemical stress, and ice crystallization, respectively. Furthermore, optimizing the recovery environment after thawing can provide additional benefits. Post-thaw supplementation with antioxidants such as glutathione (GSH) has been shown to restore intracellular redox balance and enhance hatching [43], while zona pellucida (ZP) modification strategies (e.g., enzymatic digestion or piercing) can overcome cryo-induced zona hardening that impedes blastocyst hatching [44]. These interventions complement pre-freeze strategies and are essential for restoring complete developmental competence.

In this study, we designed a sequential, multi-phase optimization strategy targeting different stages of porcine embryo cryopreservation. Specifically, we tested (i) berberine and melatonin during in vitro culture, (ii) Fe_3_O_4_ nanoparticles and AFP I during vitrification, and (iii) post-thaw interventions including GSH supplementation and zona pellucida digestion. We hypothesized that this integrated protocol would provide synergistic cryoprotection, enhancing embryo survival, developmental gene expression, and hatching efficiency to levels comparable to those of fresh controls.

## 2. Materials and Methods

### 2.1. Experimental Design

This study was designed to optimize vitrification protocols for porcine parthenogenetic embryos by integrating interventions during culture, vitrification, and post-thaw recovery. The workflow was structured in four sequential phases.

**Experiment 1** (Cryopreservation platform comparison): A benchmark comparison of three vitrification carriers (Cryotop, conventional plastic straw, and open-pulled straw (OPS)) was conducted to identify the most effective baseline method for cryopreservation.

**Experiment 2** (Culture-stage optimization): The effects of berberine (BER, 2.5 μM) and melatonin (MT, 10^−9^ M) supplementation during in vitro culture were tested to evaluate their roles in lipid reduction, antioxidant protection, and post-thaw developmental competence.

**Experiment 3** (Vitrification solution optimization): The cryoprotective effects of Fe_3_O_4_ nanoparticles (1.0%) and antifreeze protein type I (AFP I, 500 ng/mL) were tested individually and in combination during equilibration and vitrification solutions.

**Experiment 4** (Integrated Optimization and Post-Thaw Refinements): A combined approach integrating berberine and melatonin during culture with Fe_3_O_4_ and AFP-I during vitrification was evaluated. Post-thaw interventions, including glutathione (GSH, 5 mM) supplementation in culture medium and zona pellucida (ZP) digestion using protease (45 s), were further assessed for their effects on embryo hatching and quality.

This sequential design allowed progressive optimization of cryoprotection by targeting lipid metabolism, oxidative stress, ice crystal damage, and post-thaw developmental barriers.

### 2.2. Oocyte Collection and In Vitro Maturation (IVM)

Porcine ovaries were collected from slaughtered sows within 30 min postmortem and transported in sterile saline at 35 °C containing penicillin (100 IU/mL) and streptomycin (100 μg/mL). Follicular fluid was aspirated from 3–6 mm follicles using an 18-gauge needle, and cumulus-oocyte complexes (COCs) were collected and washed under a stereomicroscope. COCs were cultured in groups of 50 per well in IVM medium at 38.5 °C with 5% CO_2_ for approximately 40 h. The IVM medium consisted of TCM-199 supplemented with 10% porcine follicular fluid, 0.55 g/L glucose, 0.07 g/L cysteine, 0.1 g/L sodium pyruvate, 10 IU/mL LH, 10 IU/mL FSH, and 10 ng/mL EGF. For treatment groups, berberine (2.5 μM) and/or melatonin (10^−9^ M) were added to the IVM medium as indicated.

Mature oocytes were denuded with 1 mg/mL hyaluronidase and gentle pipetting, and those showing a first polar body and homogeneous cytoplasm were selected for parthenogenetic activation.

### 2.3. Parthenogenetic Activation of Oocytes

Selected mature oocytes were activated using a BLS fusion device with 150 V, 100 μs, 2 pulses. Activated oocytes were cultured in PZM-3 medium supplemented with 10 μg/mL cycloheximide (CHX) under 5% CO_2_ and 5% O_2_ at 38.5 °C for 4 h, followed by culture in PZM-3 culture plates for continued culture.

### 2.4. Cryopreservation Procedure

Cryopreservation was performed using various vitrification methods, including the Cryotop, Straw, and OPS methods. Five (5) embryos were sequentially incubated in vitrification solution I containing Tyrode’s lactate (TL)-HEPES-polyvinyl alcohol (TL-PVA) with 7.5% DMSO, and 7.5% EG at 41 °C for 3 min and then transferred to vitrification solution II containing TL-PVA with 16% DMSO, and 16% EG + 0.4 M sucrose for 30 s. Embryos were immediately loaded onto Cryotop, straw, and OPS carriers and plunged into liquid nitrogen within 1 min.

Experimental groups received additional supplements as follows:

**Culture stage only:** 2.5 μM berberine and/or 10^−9^ M melatonin during IVM.

**Vitrification stage only:** 1.0% Fe_3_O_4_ nanoparticles, 500 ng/mL AFP I, or their combination in Solutions I and II.

**Integrated treatment:** berberine + melatonin during IVM and Fe_3_O_4_ + AFP I during vitrification.

### 2.5. Thawing and Post-Thaw Culture

Cryotop straws were directly immersed in 2 mL thawing solution at 39 °C, and embryos were gently released (~10 s). Embryos were washed in TL-PVA, which consisted of: NaCl 7.264 g, KCl 0.237 g, NaHCO_3_ 0.168 g, KH_2_PO_4_ 0.041 g, Na-lactate 1.868 mL, MgCl_2_·6H_2_O 0.102 g, CaCl_2_·2H_2_O 0.297 g, HEPES 2.383 g, Na-pyruvate 0.022 g, sorbitol 2.186 g, PVA 1 g, and antibiotics (100 IU/mL).

The embryos were then cultured at 38.5 °C for 24 h in NCSU23 medium supplemented with 5 mM glutathione (GSH), or in standard medium after partial zona pellucida digestion with protease (45 s). A combined treatment (GSH + ZP digestion) was also evaluated, and the hatching rates were assessed.

### 2.6. Survival and Hatching Rates

Post-thaw survival was evaluated morphologically under a stereomicroscope after 24 h of culture. Embryos with intact zona pellucida and clear blastomeres were considered viable. Hatching rates were recorded at the blastocyst stage.

### 2.7. Embryo Lipid Droplet Staining

Intracellular lipid droplet staining was performed according to the methods described by Buschiazzo et al. (2017) [45] with some modifications. The embryos were rinsed three times with PBS containing 0.1% polyvinyl alcohol (PVA), then fixed with 4% paraformaldehyde for 30 min at room temperature. Following three rounds of washing with 0.1% PVA/PBS, the embryos were incubated in a 10 μg/mL Nile Red staining solution at 37 °C for 2 h. After incubation, they were examined under an inverted epifluorescence microscope (Nikon, Tokyo, Japan). The fluorescent images were statistically analyzed utilizing ImageJ software version 1.8.0 (NIH, Bethesda, MD, USA).

### 2.8. Embryo Cytoskeleton Integrity

The integrity of the embryo cytoskeleton was assessed according to Zhang et al. (2025) [46] with some modifications. The embryos were fixed with 4% paraformaldehyde at room temperature for 30 min, then washed 3 times with 0.1% TritonX-100 for 5 min each time. The embryos were placed in Tubulin-Tracker Red solution (C1050, Beyotime, Shanghai, China) at room temperature and incubated for 30 min in the dark. After that, they were washed twice in 0.1% PVA/PBS and further incubated in 1 mg/mL DAPI at 37 °C for 5 min. Then, embryos were fixed on a glass slide for examination under the confocal laser microscope (Leica, Wetzlar, Germany). ImageJ software version 1.8.0 (NIH, Bethesda, MD, USA) was used to analyze fluorescence intensity.

### 2.9. Reactive Oxygen Species (ROS) Staining

Embryo ROS staining was performed according to the method described by García-Martínez et al. (2020) [43], with some modifications. Intracellular ROS levels in embryos were quantified by labeling embryos that were washed twice in 0.1% PVA/PBS, then incubated in PVA/PBS supplemented with 5 μM 2′,7′-dichlorodihydrofluorescein diacetate (DCFH-DA) (S0033S, Beyotime) for 30 min at 38.5 °C in a humidified 5% CO_2_ air atmosphere. Then, the embryos were observed using an epifluorescence inverted microscope (Nikon, Tokyo, Japan). ImageJ software version 1.8.0 (NIH, Bethesda, MD, USA) was used to analyze fluorescence intensity.

### 2.10. ATP Measurement

ATP levels in embryos were measured according to Hara et al. (2024) [47] with some modifications. After IVM, oocytes were denuded and individually transferred into distilled water (50 μL). The adenosine triphosphate (ATP) content in individual oocytes was measured using a luciferase-based ATP determination kit (Thermo Fisher Scientific, Waltham, MA, USA). The luminescence generated by the ATP-dependent luciferin-luciferase reaction was measured using a luminometer (Spark 10 M; Tecan Japan Co., Ltd., Kanagawa, Japan). Values were normalized per embryo and expressed in pmol.

### 2.11. Apoptosis Detection (TUNEL Assay)

Embryos were fixed in 4% paraformaldehyde for 30 min, permeabilized with 0.2% Triton X-100, and stained using a TUNEL apoptosis detection kit (T2130, Solarbio, Beijing, China). Nuclei were counterstained with DAPI (5 μg/mL), and apoptotic cells were visualized using fluorescence microscopy. Quantification was performed using ImageJ.

### 2.12. Total RNA Extraction, Reverse Transcription (cDNA Synthesis), and RT-qPCR

Total RNA was prepared from pooled embryos (n = 10–15 per group) with FlysisAmp Cells-to-CT 2-Step SYBR Green Kit (Vazyme, Nanjing, China) according to the manufacturer’s protocol. Cells were processed under RNase-free conditions to minimize degradation. Suspended cells were washed once with pre-chilled 1× PBS (500 µL per 10^5^ cells). Cells were then lysed at room temperature by adding the FlysisAmp Cells-to-CT working lysis solution (FlysisAmp Cells Lysis Buffer and DNase I Solution mixed at 23:1) with gentle pipetting (8–10 times) and incubated for 5 min. Genomic DNA is removed, and RNA is released in this step. The reaction was terminated by adding the recommended volume of FlysisAmp Cells Stop Buffer, mixing gently, and then incubating for 2 min at room temperature. Lysates were used directly for reverse transcription.

Reverse transcription (cDNA synthesis) was performed using the FlysisAmp Cells-to-CT 2-Step SYBR Green Kit according to the manufacturer’s protocol. For each sample, a 20 µL RT reaction was assembled with 4 µL 5× RT Mix and 16 µL cell lysate. Mixtures were briefly mixed and centrifuged at (1480× *g*, 1 min). A no-RT negative control was prepared by substituting the 5× RT Mix with 5× No-RT Mix to monitor genomic DNA carryover. Reactions were incubated at 50 °C for 15 min, then 85 °C for 5 min, and held at 4 °C for GC-rich/structured templates. cDNA was used immediately for qPCR.

Reverse-transcriptase quantitative PCR (RT-qPCR) was performed using the FlysisAmp Cells-to-CT 2-Step SYBR Green Kit according to the manufacturer’s protocol. Gene expression was quantified using a SYBR-based, ROX-normalized master mix in 20 µL reactions containing 10 µL 2× Universal SYBR qPCR Master Mix (ROX), 0.4 µL each of forward and reverse primers (10 µM; 0.2 µM final), cDNA (5 µL), and nuclease-free water (4.2 µL) to volume. Thermal cycling was performed with an initial denaturation at 95 °C for 30 s, followed by 40 cycles of 95 °C for 10 s and 60 °C for 30 s, with instrument default melt-curve acquisition applied. The PCR primers used in the assessment are shown in Table 1.

### 2.13. Statistical Analysis

All experiments were repeated at least three times. Data were expressed as mean ± standard deviation (SD). Differences among groups were assessed by one-way ANOVA followed by Tukey’s post hoc test. A value of *p* < 0.05 was considered statistically significant.

## 3. Results

### 3.1. Comparative Efficiency of Cryopreservation Methods on Post-Thaw Viability of Parthenogenetically Activated Pig Embryos

Post-thaw survival rates of parthenogenetically activated pig embryos varied significantly among cryopreservation methods (Table 2). The Cryotop method achieved the highest survival rate (70.27 ± 7.16%), which was significantly greater (*p* < 0.05) than that of both the OPS method (65.85 ± 7.52%) and the straw method (52.94 ± 6.35%). The OPS method also produced a significantly higher survival rate compared with the straw method (*p* < 0.05).

### 3.2. Effect of Berberine on Lipid Content of Porcine Parthenogenetically Activated Pig Embryos

Berberine treatment significantly reduced embryonic lipid content, as shown in Table 3. The berberine-treated embryos had a mean fluorescence of 20.18 ± 2.51, whereas fresh controls averaged 62.63 ± 4.32.

**Figure 1:**
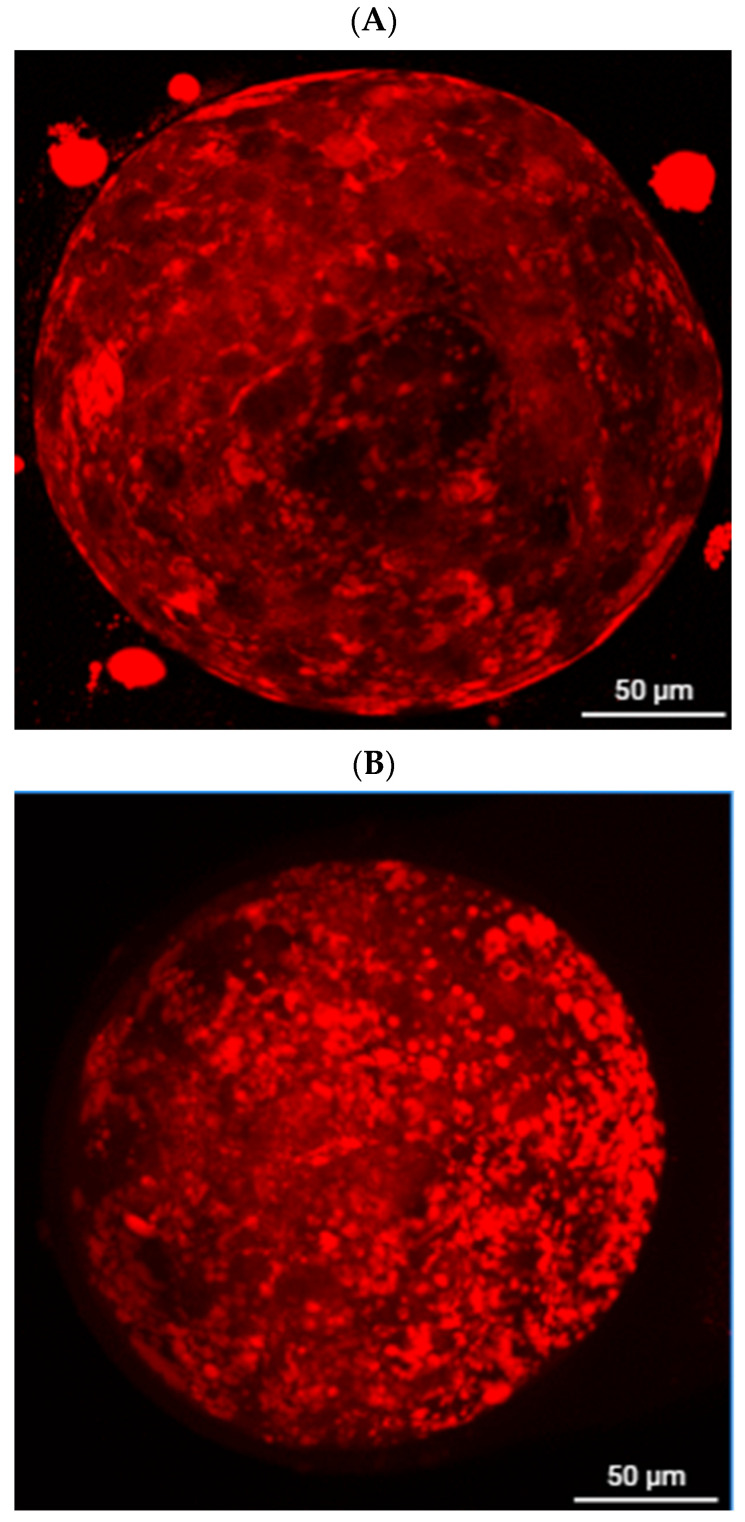
Representative images of lipid droplets stained with Nile Red (scale bar = 50 μm). (**A**): 2.5 μM Berberine group. (**B**): Fresh control group.

Figure 1 shows fluorescence micrographs of Nile Red–stained embryos. The berberine-treated embryos exhibit far fewer and smaller lipid droplets compared to controls, consistent with the quantitative reduction in lipid content shown in Table 1. This visual evidence supports the conclusion that berberine reduces embryonic fat content before freezing.

### 3.3. Berberine and Melatonin Treatments on the Freezing Effect of Parthenogenetically Activated Porcine Embryos

Table 4 shows that the combined berberine and melatonin (MT) treatment yields the best cryopreservation outcomes among the tested freeze protocols. The berberine + MT group achieved a survival rate of 87.8%, a microtubule normality of 82.7%, an ATP content of 0.32 pmol, and a hatching rate of 83.9%, all of which were significantly higher than in either the single-treatment or Cryotop-alone groups. For example, survival rates rose from 70.0% (Cryotop only) to 87.8% with berberine + MT, and the percentage of embryos with normal microtubule distribution increased from 66.7% to 82.7%. Reactive oxygen species (ROS) levels were lowest in the combined group (mean 52.85) compared to 66.32–86.32 in the other groups, indicating reduced oxidative stress. Notably, even the combined treatment did not fully restore fresh control levels (fresh embryos had 91.89% normal microtubules, 0.38 pmol ATP, and 92.59% hatching).

**Figure 2:**
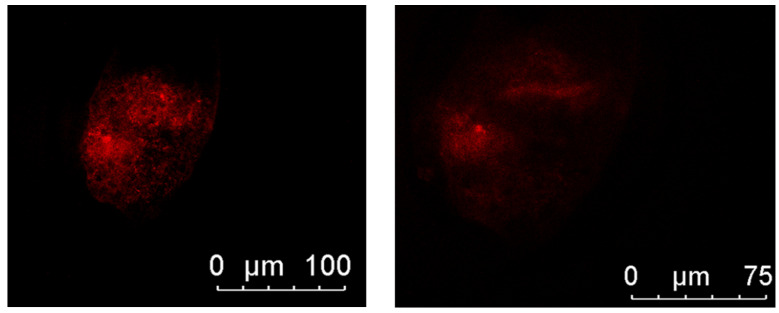
Microtubule distribution in frozen–thawed porcine embryos (normal vs. abnormal patterns).

Figure 2 illustrates microtubule organization. Embryos from the berberine + MT group show well-organized, bipolar spindle structures (typical pattern), whereas Cryotop only embryos often display disorganized (abnormal) microtubules. This visual trend aligns with the finding in Table 5 that the berberine + MT group had a significantly higher percentage of normal microtubule distribution (82.69%) compared to 66.67% in Cryotop-alone embryos.

**Figure 3:**
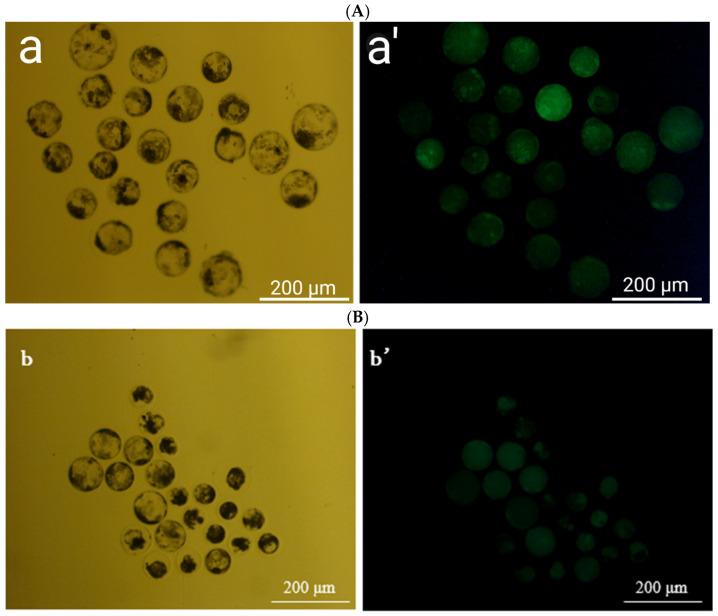
ROS staining in freeze–thawed porcine embryos. (**A**): 2.5 μM Berberine + 10^−9^ M MT. (**a**) bright-field image of the 2.5 μM Berberine + 10^−9^ M MT before ROS staining. (**a’**) fluorescence (DCFH-DA) image of the 2.5 μM Berberine + 10^−9^ M MT. (**B**): Fresh control. (**b**) bright-field image of the Fresh control embryos before ROS staining. (**b’**) fluorescence (DCFH-DA) image of the Fresh control embryos.

Figure 3 shows fluorescent ROS staining. Embryos from the berberine + MT + Cryotop group exhibit a much weaker ROS signal than those from other fresh control groups. This is consistent with the quantitative ROS values in Table 4, where the berberine + MT embryos had the lowest ROS fluorescence (52.85) compared to 66.32–86.32 in the other treatment groups.

### 3.4. Effects of Fe_3_O_4_ Nanoparticles and AFP I on the Frozen Survival Rate of Parthenogenetically Activated Porcine Embryos

Table 5 shows that supplementing both Fe_3_O_4_ nanoparticles and AFP I into the cryopreservation media markedly improves outcomes. The combined Fe_3_O_4_ + AFP I group had the highest survival (84.4%) and hatching (82.4%) rates, significantly above those of Fe_3_O_4_ or AFP I alone (~78–79%) or Cryotop alone (73.0%). Cytoskeletal integrity and ATP content are likewise most outstanding in the combined group (78.94%, 0.33 pmol) compared to ~66–74% integrity and 0.20–0.28 pmol ATP in other groups. ROS levels are dramatically lower in the Fe_3_O_4_ + AFP I embryos (40.15 fluorescence units) than in the others (~74–95), indicating reduced oxidative damage. Fresh embryos still outperform all frozen groups (survival rate of ~89%, hatching rate of ~92%, and the lowest ROS of 28.61).

### 3.5. Effect of Combined Treatment of Culture and Freezing Process on the Frozen Survival Rate of Parthenogenetically Activated Porcine Embryos

Table 6 shows that integrating all treatments at once restores embryo viability to near-normal levels. The full-combination group (berberine + MT in culture, plus Fe_3_O_4_ + AFP I in freezing) achieved 93.75% survival and 90.48% hatching rate, which were not statistically different from those of fresh controls (91.30% and 92.68%, respectively). Cytoskeleton integrity and ATP content are comparable to those of fresh samples. Significantly, ROS levels in the full-treatment embryos drop to 31.35, which is significantly lower than the 62.34–87.65 levels observed in partial treatments, indicating minimal oxidative damage. In contrast, partial treatments (either culture-only or freezing-only optimizations) yield intermediate survival rates (83.33–85.11%) and do not achieve the levels of survival seen in fresh tissue.

### 3.6. Effects of Optimization of Culture Medium After Thawing and Different Zona Pellucida Treatments on Hatching Rate of Parthenogenetically Activated Porcine Embryos

Table 7 shows that adding 5 mM GSH to the recovery culture medium significantly improves post-thaw hatching. The GSH group hatched at 82.54%, which was higher than that of the resveratrol group (76.79%) or the frozen control (72.41%). However, even with GSH, hatching remains below the fresh control level (88.5%).

Table 8 shows that mechanical or enzymatic loosening of the zona pellucida enhances hatching. The 45-s trypsin treatment produced the highest hatching rate (85.00%) among the frozen groups, significantly above the 80.77% control rate. Acidified Tyrode’s treatment (84.31%) yielded a similar benefit. Barely any embryos hatched without zona manipulation. All treated groups, however, still fell short of fresh controls (90.20%).

Table 9 shows a synergistic effect: combining trypsin digestion of the zona with 5 mM GSH in the recovery medium restores hatching to levels comparable to those in fresh. The combined treatment group had a 90.70% hatching rate, which was not statistically different from that of the fresh control (89.58%). This is a significant improvement over the 80.77% in untreated frozen controls.

### 3.7. Relative Expression Analysis of Apoptosis and Development-Related Genes

**Figure 4:**
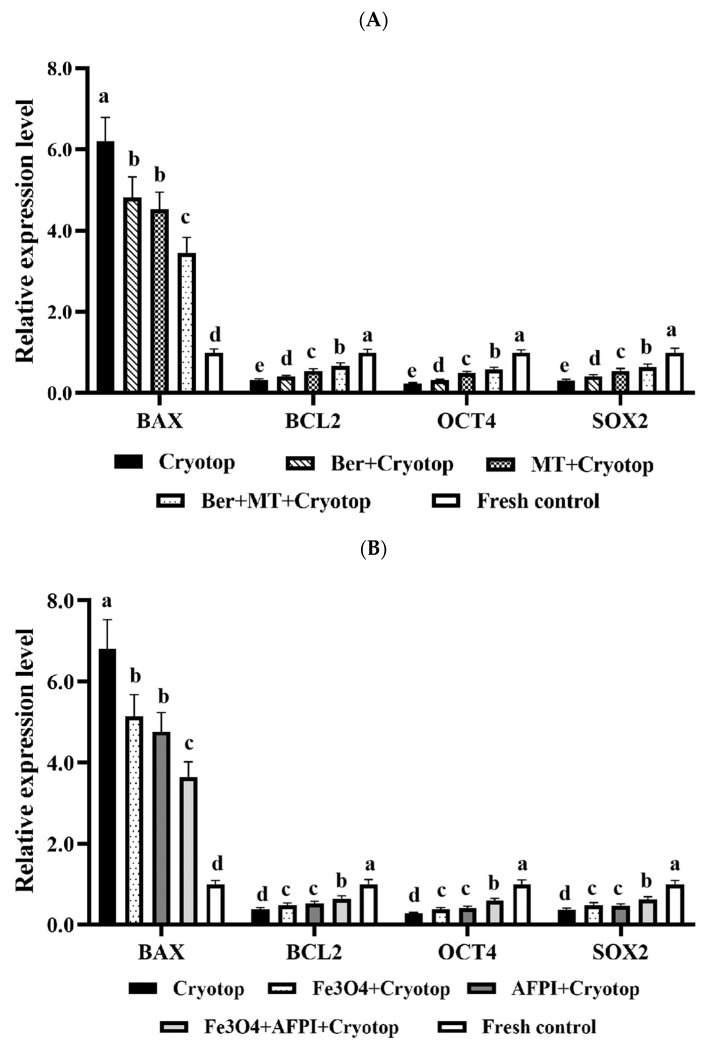

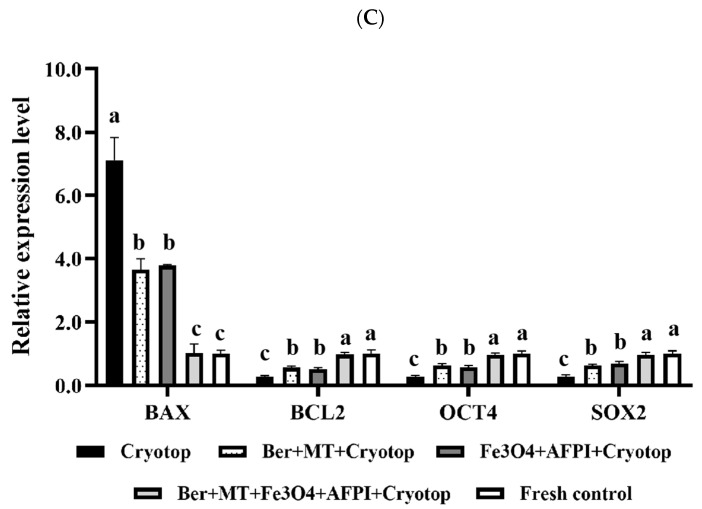
Relative Expression Analysis of Apoptosis and Development-Related Genes. (**A**): Effects of berberine (2.5 μM) and melatonin (10^–9^ M) on expression of apoptosis-related and development-related genes in frozen–thawed porcine embryos. (**B**): Effects of Fe_3_O_4_ nanoparticles (1.0%) and antifreeze protein (500 ng/mL) on expression of apoptosis-related and developmental genes in frozen–thawed porcine embryos. (**C**): Effects of combined culture (berberine + MT) and freezing (Fe_3_O_4_ + AFP I) treatments on expression of apoptosis-related and developmental genes in frozen–thawed porcine embryos. Different superscript letters indicate statistically significant differences (*p* < 0.05).

The gene expression profiles in Figure 4A indicate that the combination of berberine and MT treatment shifts the balance toward cell survival. Embryos vitrified with Cryotop alone exhibited the highest expression of pro-apoptotic BAX and the lowest levels of survival/development genes (BCL2, OCT4, SOX2). Supplementation with berberine or melatonin alone partially reduced BAX and increased the expression of BCL2, OCT4, and SOX2, but the differences remained significant compared to the fresh controls. The combined berberine and melatonin treatment resulted in a more pronounced downregulation of BAX and upregulation of BCL2, OCT4, and SOX2.

Figure 4B shows the effects of Fe_3_O_4_ nanoparticles and AFP I during vitrification on gene expression. Cryotop alone resulted in high BAX expression and low BCL2, OCT4, and SOX2 expression. The addition of Fe_3_O_4_ or AFPI individually lowered BAX and modestly increased BCL2, OCT4, and SOX2 compared to Cryotop. However, the combined Fe_3_O_4_ + AFP I treatment produced the most favorable expression pattern, with significantly reduced BAX and elevated levels of BCL2, OCT4, and SOX2, approaching those of the fresh control.

Figure 4C shows the integrated treatment of berberine, melatonin, Fe_3_O_4_, and AFP I on gene expression. Cryotop-only embryos again showed high BAX and low BCL2, OCT4, and SOX2 expression. Dual combinations (e.g., berberine + melatonin or Fe_3_O_4_ + AFP I) improved the expression profiles relative to Cryotop but remained inferior to those of fresh embryos. Notably, the whole combination (berberine + melatonin + Fe_3_O_4_ + AFP I) normalized gene expression, with BAX levels reduced to those of fresh control embryos and BCL2, OCT4, and SOX2 restored to values statistically indistinguishable from those of non-frozen embryos.

## 4. Discussion

This study systematically investigated the cryoprotective potential of combining berberine (BER), melatonin (MT), iron oxide nanoparticles (Fe_3_O_4_), and antifreeze protein I (AFP I) in porcine embryo vitrification using the Cryotop platform. The results demonstrate that while each additive alone provided measurable improvements in embryo cryo-survival, the integrated protocol, combining culture-stage supplementation with BER and MT, vitrification-stage supplementation with Fe_3_O_4_ and AFP I, and post-thaw interventions with glutathione (GSH) and zona pellucida modification, produced synergistic benefits that restored embryo viability, cytoskeletal integrity, oxidative balance, and gene expression profiles to levels comparable with fresh controls. This multi-component approach provides strong evidence that addressing cryoinjury through multiple mechanistic pathways yields more robust outcomes than targeting single factors in isolation.

The present results show that optimizing both the vitrification platform and the molecular microenvironment of porcine embryos results in consistent improvements in survival outcomes and mechanistic indicators. First, the ultra-rapid Cryotop vitrification method outperformed OPS and conventional straw methods for 2–4-cell porcine parthenotes, achieving a survival rate of 70.27 ± 7.16% compared to 65.85 ± 7.52% and 52.94 ± 6.35% with the other carriers. This finding is consistent with recent evidence that open micro-volume carriers provide superior cooling and warming kinetics and higher post-warming recovery rates, including the capacity for batch vitrification suitable for practical embryo banking and transfer [19]. These device-level advantages align with recent studies showing that in vivo–derived porcine blastocysts vitrified using Cryotop systems exhibit robust survival and only modest transcriptomic perturbations [21,48]. Together, these findings position Cryotop as the appropriate baseline method for further biochemical optimization in porcine embryo cryopreservation. Mechanistically, the advantage of the Cryotop device aligns with the well-recognized chill sensitivity of porcine embryos. This sensitivity stems from their unusually high cytoplasmic lipid content, which increases the risk of lipid phase transitions, membrane destabilization, and secondary oxidative or apoptotic injury during cooling and especially during warming [2]. Vitrification prevents bulk ice formation; however, embryos remain vulnerable to devitrification, osmotic stress, and ROS surges upon rewarming. All of these stressors are linked to apoptosis and impaired developmental competence in multiple mammalian models [1,49,50,51]. Thus, Cryotop provides the most effective baseline for vitrification of porcine embryos. However, its integration with targeted molecular interventions addressing lipid sensitivity, membrane fragility, and oxidative stress is essential to achieve consistent post-thaw developmental competence.

A key finding of this work is the lipid-lowering and antioxidant effect of berberine. Porcine embryos are notoriously sensitive to cryopreservation due to their high cytoplasmic lipid content, which undergoes phase transitions during cooling and warming, destabilizing membranes and organelles and triggering secondary ROS surges [10,11]. In this study, berberine supplementation during in vitro maturation significantly reduced lipid accumulation, as shown by Nile Red staining, and improved post-thaw survival rates. Similar findings have been reported in bovine oocytes, where low-dose berberine reduced lipid droplets, decreased ROS, and enhanced blastocyst development following vitrification [29]. The mechanism likely involves the ability of berberine to scavenge ROS directly and modulate lipid metabolism through mitochondrial and ER pathways [30]. Notably, the protective effects of berberine are dose-dependent, with beneficial outcomes at micromolar concentrations but toxicity at higher doses [52]. Our use of 2.5 μM was therefore appropriate, as it conferred protection without impairing development.

Melatonin supplementation provided additional benefits by reducing oxidative stress and improving mitochondrial stability. When combined with berberine, the effects were synergistic, resulting in significantly higher survival, ATP content, cytoskeletal integrity, and hatching rates, as well as lower ROS and apoptosis levels compared to Cryotop alone. Melatonin is a well-characterized cryoprotectant in gametes and embryos, acting as both a potent free radical scavenger and a regulator of mitochondrial function [31]. In porcine oocytes, melatonin enhances GSH content, reduces ROS, improves mitochondrial distribution, and promotes cleavage and blastocyst formation [32]. Our findings are consistent with these protective roles and suggest that berberine and melatonin act through complementary mechanisms; berberine mitigates lipid-associated ROS generation, while melatonin regulates mitochondrial redox balance and apoptosis signaling. Together, these mechanisms explain the superior performance of the dual treatment group compared to single additives.

In addition to culture-stage antioxidants, the incorporation of Fe_3_O_4_ nanoparticles and AFP I during vitrification also enhanced cryoprotection by targeting biophysical and structural aspects of cryoinjury. Fe_3_O_4_ nanoparticles possess superparamagnetic properties that enhance thermal conductivity, reducing localized ice formation and improving rewarming kinetics. They may also interact with plasma membranes to reinforce structural integrity. In mouse oocytes, Fe_3_O_4_ nanoparticles improved nuclear maturation and blastocyst development while reducing apoptosis and ultrastructural damage during vitrification [33]. Our findings align with these results, as embryos treated with Fe_3_O_4_ nanoparticles showed improved cytoskeletal integrity and ATP content compared with those treated with Cryotop alone. AFP I, on the other hand, inhibits ice nucleation and recrystallization, thereby preventing mechanical and osmotic damage during thawing [35,39]. Previous studies have demonstrated AFP-mediated cryoprotection in sheep [40] and bovine embryos [41], consistent with our observation that AFP I significantly improved survival and hatching rates in porcine embryos. Importantly, the combination of Fe_3_O_4_ and AFP I produced superior outcomes compared to either agent alone, highlighting the complementary mechanisms of thermal stabilization and ice inhibition.

The integrated treatment, which combines berberine, melatonin, Fe_3_O_4_, and AFP I, was the most effective strategy, restoring embryo survival and hatching rates to levels not statistically different from those of fresh controls. ROS levels were significantly reduced, ATP content was restored, and cytoskeletal organization was preserved, and post-thaw embryos exhibited gene expression patterns similar to those of fresh embryos. These results demonstrate clear synergy among the additives, which together address the multifactorial nature of cryoinjury. This synergy likely reflects the convergence of orthogonal mechanisms: berberine and melatonin mitigate oxidative and apoptotic injury, Fe_3_O_4_ nanoparticles enhance thermal dynamics and structural stability, and AFP I prevents ice-induced damage. While vitrification prevents bulk ice formation, embryos remain vulnerable to lipid phase transitions, osmotic stress, and oxidative damage during warming [2]. The combined protocol effectively mitigated these stressors, confirming that simultaneous targeting of oxidative stress, ice crystallization, and membrane fragility is required for optimal embryo cryoprotection.

Post-thaw interventions further contributed to restoring developmental competence. Supplementation of the recovery medium with glutathione improved hatching rates, consistent with its central role in intracellular redox homeostasis and in detoxifying peroxides. Similar findings have shown that GSH supplementation restores redox balance and enhances hatching in vitrified porcine embryos [43]. Cryo-induced zona pellucida hardening is another significant barrier to embryo development. Enzymatic digestion or piercing of the zona has been shown to restore hatching by overcoming this barrier [44]. Our data demonstrate that combining GSH supplementation with zona digestion restored hatching efficiency to levels comparable to those of fresh controls, confirming the importance of optimizing both pre-freeze and post-thaw conditions for maximal embryo competence.

At the molecular level, gene expression profiling provided critical mechanistic insight. Cryotop vitrification alone upregulated the pro-apoptotic gene BAX and downregulated the pluripotency-associated genes BCL2, OCT4, and SOX2, indicating activation of apoptosis and suppression of pluripotency. This finding is consistent with previous studies, which have reported that cryopreservation induces oxidative stress, apoptosis, and developmental arrest in porcine embryos [13]. Supplementation with berberine or melatonin alone partially normalized these expression patterns, while the combined treatment more strongly reduced BAX and increased BCL2, OCT4, and SOX2. Similarly, Fe_3_O_4_ nanoparticles and AFP I partially improved expression profiles, with the combined group showing more potent effects. Most importantly, the integrated treatment with berberine, melatonin, Fe_3_O_4_, and AFP-I fully restored gene expression to levels indistinguishable from those of fresh embryos. The downregulation of BAX and concurrent upregulation of BCL2 confirmed an anti-apoptotic shift, while the restoration of OCT4 and SOX2 expression indicated preservation of pluripotency and developmental potential. These findings suggest that the multi-component protocol not only prevented structural and oxidative damage but also preserved the transcriptional and epigenetic integrity necessary for normal embryogenesis.

**Figure 5:**
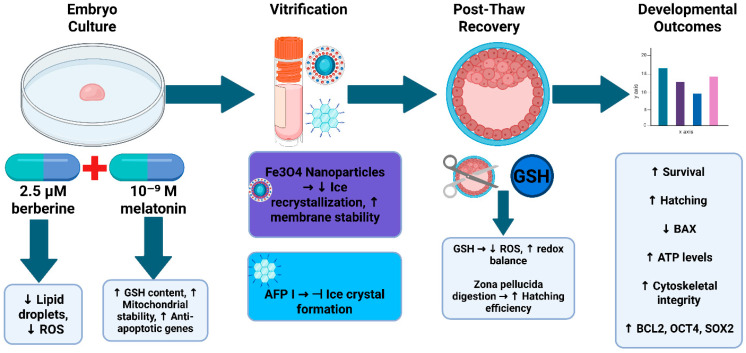
Schematic Model of the Integrated Antioxidant, Nanoparticle, and Antifreeze Protein Strategy for Enhancing Cryosurvival and Developmental Competence of Porcine Embryos. This schematic illustrates how berberine and melatonin supplementation during embryo culture reduces lipid accumulation, oxidative stress, and apoptosis; how Fe_3_O_4_ nanoparticles and antifreeze protein I during vitrification inhibit ice crystal formation, enhance membrane stability, and improve thermal protection; and how post-thaw interventions with glutathione and zona pellucida digestion restore redox balance and promote hatching. Together, these multi-stage interventions synergistically improve cryosurvival, mitochondrial function, cytoskeletal integrity, and gene expression profiles, ultimately supporting enhanced developmental competence and fertility-related outcomes. Created in BioRender. EDNA, U. (2025) https://BioRender.com/qefazjq, accessed on 20 November 2025.

Mechanistically, this study supports a multi-layered model of cryoprotection. Berberine and melatonin mitigate ROS generation, lipid peroxidation, and apoptotic activation, while stabilizing mitochondria. Fe_3_O_4_ nanoparticles enhance thermal conductivity and membrane stability, and AFP I inhibits ice recrystallization and mechanical damage during thawing. Post-thaw supplementation with GSH restores redox balance, and zona pellucida modification overcomes structural barriers to hatching. Collectively, these interventions act on orthogonal pathways, explaining the robust synergy observed in the integrated treatment. A schematic summary of these multi-stage protective mechanisms is presented in Figure 5.

Despite these promising outcomes, limitations should be acknowledged. Parthenogenetic embryos were used as a model, which may not fully replicate in vivo fertilized embryos. Further validation in in vivo–derived embryos and across multiple pig breeds is warranted. Functional fertility trials, including embryo transfer and assessment of offspring development, are necessary to confirm translational efficacy. Accordingly, this study cannot determine whether the integrated supplementation strategy would affect pregnancy or birth rates after embryo transfer, and future in vivo reproductive trials will be required to evaluate these outcomes. Additionally, while Fe_3_O_4_ nanoparticles demonstrated strong protective effects, their potential toxicity and residual retention require further investigation before widespread application. Finally, while gene expression profiling revealed protective shifts, embryos are transcriptionally limited; thus, proteomic and metabolomic analyses would provide deeper mechanistic insight.

The implications of these findings are significant. For agriculture, improved porcine embryo cryopreservation will accelerate genetic improvement, enable secure international germplasm exchange, and aid in the conservation of rare breeds [3,4]. While these findings suggest strong potential for future application in commercial pig production systems, additional in vivo studies, including long-term storage assessments and embryo-transfer trials, are needed to confirm that the integrated cryoprotective strategy can consistently prevent functional degradation associated with extended freezing durations. For biomedicine, efficient preservation of genetically engineered pig embryos is critical for developing disease models and advancing xenotransplantation [6]. More broadly, this study establishes a mechanistic framework for the rational design of cryoprotectant regimens that can be adapted for use in other species. By integrating antioxidants, nanoparticles, antifreeze proteins, and post-thaw refinements, the protocol described here provides a paradigm for next-generation embryo cryopreservation strategies.

Looking ahead, the advantages of this multi-component vitrification strategy, particularly its ability to restore embryo quality to near-fresh levels, indicate substantial potential for future industrial application in large-scale porcine breeding programs, germplasm banking, and genetic improvement platforms. Although the present work focused on a porcine model, several underlying principles, including mitigating oxidative stress, improving thermal conductivity, and inhibiting ice recrystallization, may be relevant to advancing human-assisted reproductive technologies. Further research should therefore include long-term storage studies, embryo-transfer trials to assess pregnancy and offspring outcomes, and eventual cross-species translational investigations to determine whether these mechanisms can be safely and effectively adapted for human clinical use.

In conclusion, this work demonstrates that a multi-component strategy integrating berberine, melatonin, Fe_3_O_4_ nanoparticles, and AFP I during Cryotop vitrification, complemented by post-thaw interventions, synergistically protects porcine embryos against cryoinjury. By simultaneously targeting oxidative stress, mitochondrial dysfunction, cytoskeletal disruption, and ice recrystallization, the protocol restored embryo survival and developmental competence to levels comparable to those of fresh controls. These findings advance both the mechanistic understanding and practical application of cryopreservation in pigs, providing a roadmap for improving germplasm preservation across agricultural and biomedical contexts.

## 5. Conclusions

This study demonstrates that cryoinjury in porcine embryos can be effectively mitigated by a multifaceted Cryotop vitrification strategy integrating antioxidant, nanomaterial, and protein-based interventions. Specifically, the antioxidants berberine and melatonin play pivotal mechanistic roles during oocyte maturation: berberine reduces cytoplasmic lipid accumulation and mitigates ROS generation. In contrast, melatonin enhances mitochondrial function and reinforces anti-apoptotic signaling, ultimately leading to improved post-thaw survival and developmental competence of the embryos. In parallel, incorporating Fe_3_O_4_ nanoparticles into the vitrification medium improves thermal conductivity and fortifies membrane integrity. At the same time, antifreeze protein I (AFP I) inhibits ice nucleation and recrystallization, collectively protecting embryos from thermal and mechanical cryodamage. By targeting these complementary pathways, the combined treatment yields a robust synergistic cryoprotective effect. Embryos vitrified with berberine, melatonin, Fe_3_O_4_, and AFP I exhibit significantly higher post-thaw survival (cryosurvival) and hatching rates, statistically indistinguishable from fresh controls, along with restored ATP levels, preserved cytoskeletal structure, and minimal ROS-induced damage. Moreover, post-thaw interventions, including glutathione supplementation to reestablish redox balance and partial zona pellucida digestion to alleviate hatching constraints, further promote near-fresh recovery of embryo function. Remarkably, embryos receiving the full regimen display gene expression profiles characteristic of fresh embryos, with pro-apoptotic BAX suppressed and pro-survival/pluripotency markers (BCL2, OCT4, SOX2) restored to baseline levels—indicative of fully preserved developmental potential. Collectively, these findings underscore that concurrent targeting of oxidative stress, mitochondrial dysfunction, and ice-crystal-induced injury can maintain the viability and developmental competence of vitrified porcine embryos. This multi-component approach provides a mechanistic framework for next-generation cryopreservation strategies in both agricultural and biomedical applications, potentially extending to other species.

## Figures and Tables

**Table 1:** Primers used for the RT-qPCR.

Gene	Primer	Accession Number
*BAX*	F: TCTGAGCAGATCATGAAGACAGG R: GCAGCTCCATGTTACTGTCC	XM_003127290.5
*BCL2*	F: AGTTCGGTGGGGTCATGTG R: ATACAGCTCCACAAAGGCATC	XM_021099593.1
*OCT4*	F: CTATGACTTCTGCGGAGGGAT R: TTTGATGTCCTGGGACTCCTCG	NM_001113060.1
*SOX*2	F: ATGGGCTCAGTGGTCAAGTC R: AGAGAGGCAGTGTACCGTTG	NM_001123197.1
*GAPDH*	F: CGATGGTGAAGGTCGGAGTG R: TACGACCACCCCATCCAAGT	XM_021091114.1

**Table 2:** Effects of Different Cryopreservation Methods on the Survival Rate of Parthenogenetically Activated Pig Embryos.

Group	Post-Thaw Survival Rate (%)
Straw Method	52.94 ± 6.35 ^c^ (18/34)
OPS Method	65.85 ± 7.52 ^b^ (27/41)
Cryotop	70.27 ± 7.16 ^a^ (26/37)

Different superscript letters indicate statistically significant differences (*p* < 0.05).

**Table 3:** Effect of 2.5 μM berberine on lipid content of porcine parthenogenetic embryos.

Group	Embryo Lipid Content (Fluorescence Intensity/Embryo)
2.5 μM Berberine group	20.18 ± 2.51 ^b^ (n = 30)
Fresh control group	62.63 ± 4.32 ^a^ (n = 30)

Different superscript letters indicate statistically significant differences (*p* < 0.05).

**Table 4:** Effect of 2.5 μM berberine and 10^−9^ M melatonin on cryopreservation outcomes of porcine parthenogenetic embryos.

Group	Survival Rate (%)	Microtubule Normal (%)	ROS (Fluorescence/Embryo)	ATP (pmol/Blastocyst)	Hatching Rate (%)
Cryotop only	70.00 ± 7.47 ^c^ (245/350)	66.67 ± 7.45 ^e^ (22/33)	86.32 ± 6.78 ^a^ (n = 30)	0.21 ± 0.03 ^d^ (n = 30)	70.73 ± 6.89 ^d^ (29/41)
2.5 μM Berberine + Cryotop	78.05 ± 8.46 ^b^ (224/287)	72.73 ± 6.19 ^d^ (24/33)	74.68 ± 7.15 ^b^ (n = 32)	0.26 ± 0.03 ^c^ (n = 30)	76.19 ± 8.16 ^c^ (32/42)
10^−9^ M Melatonin + Cryotop	81.09 ± 7.44 ^b^ (210/259)	77.14 ± 7.26 ^c^ (27/35)	66.32 ± 5.42 ^c^ (n = 34)	0.27 ± 0.03 ^c^ (n = 30)	77.55 ± 7.68 ^c^ (38/49)
2.5 μM Berberine + 10^−9^ M MT + Cryotop	87.80 ± 6.77 ^a^ (252/287)	82.69 ± 8.78 ^b^ (43/52)	52.85 ± 5.14 ^d^ (n = 33)	0.32 ± 0.04 ^b^ (n = 30)	83.90 ± 8.53 ^b^ (73/85)
Fresh control (no freeze)	—	91.89 ± 8.32 ^a^ (34/37)	25.67 ± 3.57 ^e^ (n = 31)	0.38 ± 0.05 ^a^ (n = 30)	92.59 ± 8.16 ^a^ (50/54)

Different superscript letters indicate statistically significant differences (*p* < 0.05).

**Table 5:** Effect of Fe_3_O_4_ nanoparticles (1.0%) and antifreeze protein (AFP, 500 ng/mL) on survival of frozen porcine parthenotes.

Group	Survival Rate (%)	Hatching Rate (%)	Cytoskeleton Integrity (%)	ROS (Fluorescence/Embryo)	ATP (pmol/Blastocyst)
Cryotop only	72.97 ± 5.65 ^c^ (197/270)	71.15 ± 7.36 ^d^ (35/52)	66.03 ± 7.36 ^d^ (35/53)	94.72 ± 10.36 ^a^ (n = 30)	0.20 ± 0.03 ^d^ (n = 30)
1.0% Fe_3_O_4_ + Cryotop	78.05 ± 6.48 ^b^ (192/246)	76.92 ± 8.52 ^c^ (40/52)	73.68 ± 6.28 ^c^ (28/38)	76.32 ± 7.41 ^b^ (n = 30)	0.28 ± 0.01 ^c^ (n = 30)
500 ng/mL AFP I + Cryotop	79.07 ± 8.42 ^b^ (204/258)	75.00 ± 6.28 ^c^ (42/56)	73.52 ± 7.16 ^c^ (25/34)	74.28 ± 6.18 ^b^ (n = 30)	0.27 ± 0.02 ^c^ (n = 30)
1.0% Fe_3_O_4_ + 500 ng/mL AFP I + Cryotop	84.44 ± 9.64 ^a^ (228/270)	82.36 ± 6.72 ^b^ (42/51)	78.94 ± 8.34 ^b^ (30/38)	40.15 ± 8.32 ^c^ (n = 30)	0.33 ± 0.04 ^b^ (n = 30)
Fresh control (no freeze)	—	88.89 ± 9.16 ^a^ (32/36)	92.30 ± 8.68 ^a^ (36/39)	28.61 ± 3.05 ^d^ (n = 30)	0.43 ± 0.02 ^a^ (n = 30)

Different superscript letters indicate statistically significant differences (*p* < 0.05).

**Table 6:** Effects of combined culture (2.5 μM berberine + 10^−9^ M MT) and freezing (1.0% Fe_3_O_4_ + 500 ng/mL AFP I) treatments on survival of porcine parthenotes.

Group	Survival Rate (%)	Hatching Rate (%)	Cytoskeleton Integrity (%)	ROS (Fluorescence/Embryo)	ATP (pmol/Blastocyst)
Cryotop only	71.11 ± 4.44 ^c^ (160/225)	68.42 ± 8.37 ^c^ (26/38)	69.23 ± 6.48 ^c^ (27/39)	87.65 ± 8.34 ^a^ (n = 30)	0.22 ± 0.03 ^c^ (n = 30)
Berberine + MT + Cryotop	83.33 ± 8.42 ^b^ (140/168)	74.36 ± 6.48 ^b^ (29/39)	73.68 ± 8.42 ^b^ (28/38)	64.25 ± 6.46 ^b^ (n = 30)	0.28 ± 0.02 ^b^ (n = 30)
Fe_3_O_4_ + AFP + Cryotop	85.11 ± 8.64 ^b^ (160/188)	75.56 ± 7.46 ^b^ (34/45)	76.32 ± 7.46 ^b^ (29/38)	62.34 ± 5.19 ^b^ (n = 30)	0.31 ± 0.03 ^b^ (n = 30)
Berberine + MT + Fe_3_O_4_ + AFP + Cryotop	93.75 ± 8.64 ^a^ (180/192)	90.48 ± 9.45 ^a^ (38/42)	90.70 ± 8.16 ^a^ (39/43)	31.35 ± 6.31 ^c^ (n = 30)	0.39 ± 0.04 ^a^ (n = 30)
Fresh control (no freeze)	91.30 ± 8.68 ^a^ (42/46)	92.68 ± 8.46 ^a^ (38/41)	29.32 ± 3.42 ^c^ (n = 30)	—	0.41 ± 0.05 ^a^ (n = 30)

Different superscript letters indicate statistically significant differences (*p* < 0.05).

**Table 7:** Effect of post-thaw culture medium supplementation on hatching of frozen porcine parthenotes.

Group	Hatching Rate (%)
1 μM Resveratrol	76.79 ± 7.64 ^b^ (43/56)
5 mM Glutathione (GSH)	82.54 ± 8.42 ^b^ (52/63)
Cryotop frozen control	72.41 ± 7.48 ^c^ (42/58)
Fresh control (no freeze)	88.52 ± 8.18 ^a^ (54/61)

Different superscript letters indicate statistically significant differences (*p* < 0.05).

**Table 8:** Effect of zona pellucida treatments on hatching of frozen porcine parthenotes.

Treatment	Hatching Rate (%)
Trypsin 45 s (partial digestion)	85.00 ± 8.46 ^b^ (34/40)
Acid Tyrode’s solution 45 s	84.31 ± 7.42 ^bc^ (43/51)
Zona piercing	82.22 ± 8.54 ^c^ (37/45)
Cryotop frozen control	80.77 ± 7.15 ^c^ (42/52)
Fresh control (no freeze)	90.20 ± 8.18 ^a^ (46/51)

Different superscript letters indicate statistically significant differences (*p* < 0.05).

**Table 9:** Combined post-thaw GSH supplementation and zona digestion on hatching of frozen porcine parthenotes.

Treatment	Hatching Rate (%)
Trypsin 45 s + 5 mM GSH (post-thaw)	90.70 ± 9.52% ^a^ (39/43)
Cryotop frozen control	80.77 ± 8.64% ^b^ (42/52)
Fresh control (no freeze)	89.58 ± 8.18% ^a^ (43/48)

Different superscript letters indicate statistically significant differences (*p* < 0.05).

## Data Availability

The original contributions presented in the study are included in the article; further inquiries can be directed to the corresponding authors.

## Abbreviations

The following abbreviations are used in this manuscript:

AFP I	Antifreeze Protein I
ATP	Adenosine Triphosphate
ROS	Reactive Oxygen Species
BAX	Bcl-2–Associated X Protein
BCL2	B-Cell Lymphoma 2
OCT4	Octamer-Binding Transcription Factor 4
SOX2	SRY-Box Transcription Factor 2
BER	Berberine
GSH	Glutathione
ZP	Zona Pellucida
COCs	Cumulus-Oocyte Complexes
CHX	Cycloheximide
OPS	open-pulled straw
TL	Tyrode’s lactate (in TL-PVA solutions)
TL-PVA	TL-HEPES-polyvinyl alcohol solution
PVA	Polyvinyl Alcohol
DCFH-DA	2′,7′-dichlorodihydrofluorescein diacetate (ROS probe)
RT-qPCR	Reverse-Transcriptase quantitative PCR
PBS	Phosphate-buffered saline (wash buffer)
DAPI	DNA-binding nuclear stain
cDNA	Complementary DNA
RNase/DNase I	Ribonuclease/Deoxyribonuclease I (used in RNA prep)
LH	Luteinizing hormone
FSH	Follicle-stimulating hormone
EGF	Epidermal growth factor
